# Association between constipation and risk of stroke: a systematic review and meta-analysis

**DOI:** 10.3389/fneur.2025.1594535

**Published:** 2025-08-13

**Authors:** Feng Tang, Tianjun Zhao, Peiwen Dong, Kaidi Sun, Xiaobin Sun, Qiong Wang

**Affiliations:** ^1^Department of Gastroenterology, The Affiliated Hospital of Southwest Jiaotong University, The Third People's Hospital of Chengdu, Chengdu, China; ^2^Department of Cardiology, The Affiliated Hospital of Southwest Jiaotong University, The Third People's Hospital of Chengdu, Cardiovascular Disease Research Institute of Chengdu, Chengdu, China

**Keywords:** constipation, stroke, ischemic stroke, gut-brain axis, meta-analysis

## Abstract

**Objective:**

This systematic review and meta-analysis aimed to evaluate the association between constipation and stroke risk, with subgroup analyses exploring effects on stroke subtypes.

**Methods:**

We systematically searched PubMed, Web of Science, and Cochrane Library until February 2025. Published studies reporting adjusted odds ratios (ORs), hazard ratios (HRs), or relative risks (RRs) for stroke in constipated versus non-constipated individuals were included. A random-effects model was used to pool effect estimates, with heterogeneity assessed via the chi-square test based on Cochrane Q statistics. Subgroup evaluations were conducted for stroke type (ischemic/hemorrhagic), region, study design, and sex.

**Results:**

Thirteen studies involving 684,123 constipation cases and 5,223,378 controls were analyzed. Constipation was associated with a 23% increased stroke risk (pooled OR = 1.23, 95% CI: 1.10–1.36, *I*^2^ = 96.51%). Subgroup analyses revealed a stronger association with ischemic stroke (OR = 1.39, 95% CI: 1.19–1.60, *I*^2^ = 96.64%) but not hemorrhagic stroke (OR = 1.03, 95% CI: 0.80–1.26, *I*^2^ = 78.38%). Notably, constipation showed no stroke risk elevation in women (OR = 1.00, 95% CI: 0.92–1.07, *I*^2^ = 0%).

**Conclusion:**

Our meta-analysis identified constipation as a risk factor for ischemic stroke, but not hemorrhagic stroke. These findings underscore constipation as a modifiable risk factor in ischemic stroke management, warranting further mechanistic and interventional studies.

**Systematic Review Registration:**

PROSPERO 2024; https://www.crd.york.ac.uk/PROSPERO/view/CRD42024615237.

## 1 Introduction

Constipation, affecting over 10% of the global population, represents a prevalent manifestation of gastrointestinal dysfunction and a critical worldwide epidemiological challenge (1). It is characterized by unsatisfactory defecation as a result of infrequent stools, difficult stool passage, or a combination of both mechanisms (2). According to the cause, constipation may be classified as primary or secondary. Secondary constipation arises from diverse etiological factors, including anatomical abnormalities (anorectal and colonic pathologies), dietary influences, pharmacological agents (notably opioids), and other underlying conditions (3). Resolution of secondary constipation—whether acute or chronic—requires addressing the primary etiology, potentially necessitating stool disimpaction, discontinuation of causative medications, or correction of structural colonic pathology. In contrast, primary chronic constipation represents a symptom-based disorder attributable to dysregulation of colonic motility, incoordination of anorectal neuromuscular function, and impaired brain–gut axis signaling (4). Based on distinct pathophysiological mechanisms, constipation is subcategorized into three subtypes: normal-transit constipation, slow-transit constipation, and rectal evacuation disorders, including dyssynergic and inadequate defecatory propulsion (5–7). Constipation may increase the risk of multiple diseases including Parkinson’s disease, multiple sclerosis, ischemic heart disease, depression or anxiety, and so on (8). Even among the diseases of gut-brain interactions, only constipation was significantly associated with mortality, while other diseases, including irritable bowel syndrome, chronic diarrhea, dyspepsia and abdominal pain, did not reduce survival rates (9). Stroke, as the most common severe neurological disorder, is frequently complicated by gastrointestinal dysmotility, with clinical studies reporting a constipation prevalence of 29–79% among affected individuals (10–12).

Although post-stroke constipation is a well-recognized complication, emerging clinical studies are now investigating the reverse association: whether constipation independently elevates stroke risk through mechanisms such as gut dysbiosis, gut-brain axis dysregulation, or shared vascular risk profiles (13–17). A previous meta-analysis (including 8 studies) indicated that constipation was associated with a higher risk of stroke. However, the study data included in this meta-analysis were limited to May 2024, and its subgroup analyses were limited to ischemic and mixed stroke subtypes, excluding hemorrhagic stroke (18). Some new studies involving relationship between constipation and risk of stroke were published from then on (14, 15). To obtain a more comprehensive estimate of the putative influence of the constipation on stroke, we performed a meta-analysis of published studies to determine the association between constipation and risk of stroke.

## 2 Methods

### 2.1 Search strategy

The meta-analysis was conducted in strict accordance with the PRISMA (Preferred Reporting Items for Systematic Reviews and Meta-Analyses) guidelines (19). The study protocol has been registered in the International Prospective Register of Systematic Reviews (PROSPERO - registration number: CRD42024615237). Based on the PRISMA guidelines, we performed a systematic search of online databases (PubMed, Web of Science, and the Cochrane Library) before February 2025. The following search terms were used in our search strategies: (‘stroke’ or ‘brain ischemic’ or ‘transient brain ischemia’ or ‘cerebra arterial disease’ or ‘CVA’ or ‘non-ischemic stroke’ or ‘ischemic stroke’ or ‘cerebrovascular accident’ or ‘cerebrovascular disorders’ or ‘intracranial artery disease’ or ‘intracerebral hemorrhage’ or ‘cardiovascular disease’ or ‘cardiovascular diseases’ or ‘CVD’ or ‘cardiovascular events’) AND (‘constipation’ or ‘Bowel Movement Frequency’).

### 2.2 Inclusion criteria

The identified studies were included for the meta-analysis if they fulfilled the following criteria: (1) eligibility will be restricted to peer-reviewed published literature, encompassing both observational investigations (cross-sectional, cohort, and case–control designs) and randomized controlled trials (RCTs); (2) based on humans; (3) the exposure was constipation, which was diagnosed according to clear diagnostic criteria; (4) each study must include one group of constipated patients and another group of non-constipated patients, and must provide an effect estimate representing the association between constipation and stroke risk in the form of a relative hazard ratio (RR), odds ratio (OR), or hazard ratio (HR), with a corresponding 95% confidence interval (CI), or sufficient raw data to calculate an estimate.

### 2.3 Exclusion criteria

In the process of literature screening, the following items of research were excluded: (1) case reports, conference abstracts, review papers, editorials, commentaries; (2) non-English literature; (3) articles without sufficient data to assess the association between constipation and stroke risk.

### 2.4 Data abstraction and quality assessment

All data were independently extracted by two reviewers using a standardized data collection table. Discrepancies in data extraction were resolved by consensus. We extracted the following data from each study: first author’s name, publication year, country, study design, participants, method of collecting constipation symptom data, definition of constipation, number of case and control groups, length of follow-up, and adjustment for covariates. The quality of each study was assessed by the Newcastle-Ottawa Scale (NOS), a standard commonly used to assess quality in cohort studies (20). The scoring system consisted of three parts: population selection, comparability between groups, and exposure factors. Results ranged from 0 to 9, with higher scores indicating better quality of the method.

### 2.5 Statistical analysis

Results from cohort studies are usually expressed in terms of relative risk (RR) or hazard ratio (HR), while results from nested case–control studies and cross-sectional studies are usually expressed as odds ratios (OR). When analyzing data, whenever possible, multivariate adjusted outcome data (all expressed as OR and 95% CI) were used. We transformed these values in each study by using their natural logarithms, and calculated standard error based on these logarithmic values and their corresponding 95% CIs. A summary of pooled-effect estimates and corresponding 95% CI were obtained by using a random-effects model, which takes into account both within-study and between-study variabilities. Heterogeneity among studies was assessed using the chi-square test based on Cochrane Q statistics at *p* < 0.05 level of significance, and quantification of heterogeneity was made by the *I*^2^ metric, which describes the estimated percentage of variability for effects due to differences rather than chance. When *I*^2^ > 50%, there was significant statistical heterogeneity in this study. To explore possible explanations for homogeneity and test the robustness of the association between constipation and risk of stroke, we conducted sensitivity analyses and subgroup analyses by study design type, stroke type, region, definition of constipation, and gender. To identify possible sources of heterogeneity, a meta-regression analysis was conducted by including covariates such as study design, region, stroke type, definition of constipation, and gender composition. A funnel plot was used to investigate possible publication bias, true heterogeneity and other methodological irregularities. We also performed the Begg’s test and Egger’s test to estimate a possible asymmetry of the funnel plot. A nonparametric trim-and-fill analysis of publication bias were used to address potential bias. A sensitivity analysis was performed, based on excluding one study at a time, to examine the impact of each exclusion on the pooled estimates and variances of the included studies. All statistical analyses were achieved using Stata18.0 software (Stata Corp, College Station, TX). *p* values were 2-sided and *p* < 0.05 was considered statistically significant.

## 3 Results

### 3.1 Study characteristics

**Figure 1:**
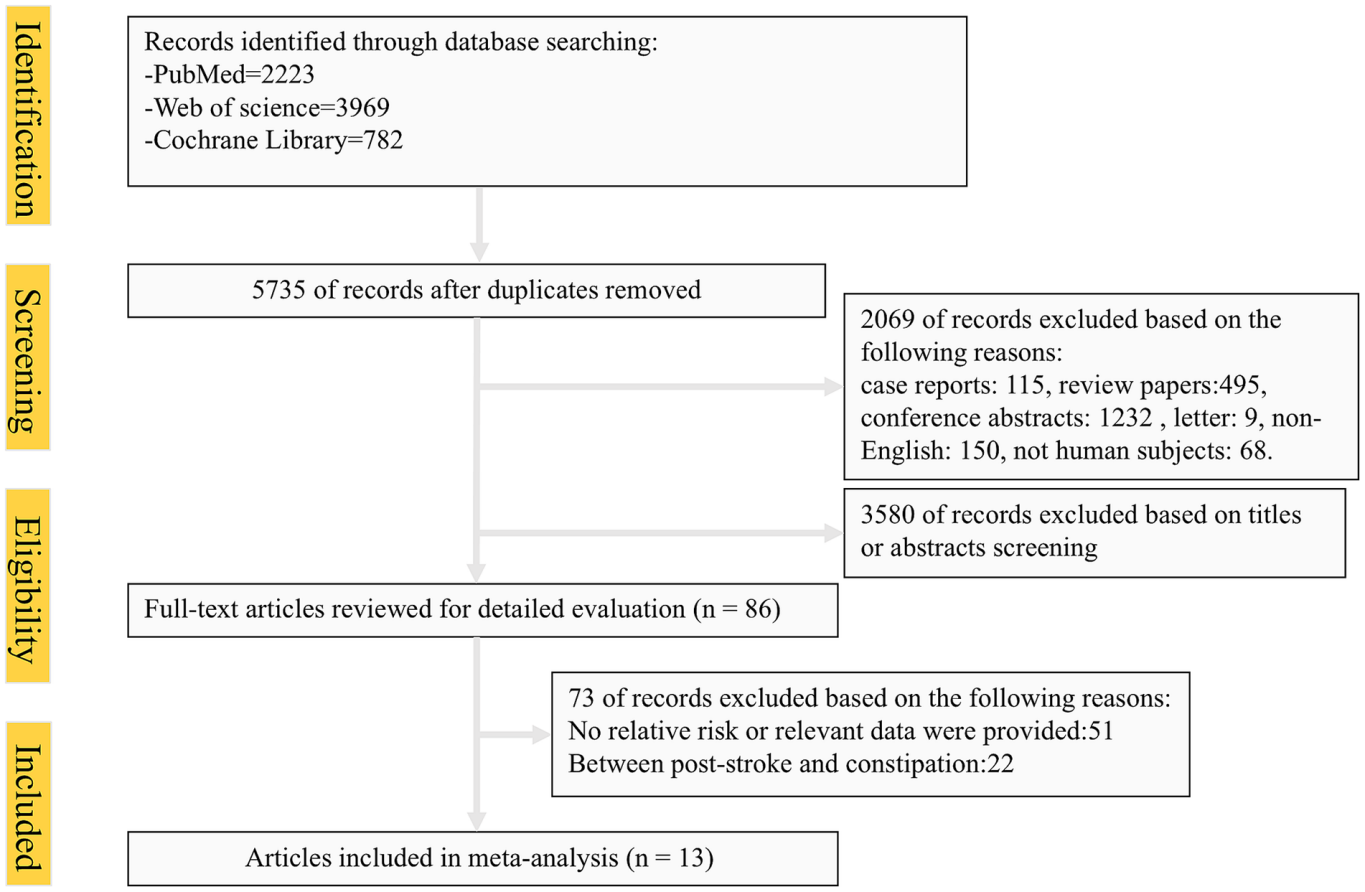
Flow chart of selected articles.

The systematic search identified 6,974 articles from online databases that were subsequently examined on title and abstract. Figure 1 shows the stages in obtaining studies for inclusion in the review. Finally, a total of thirteen articles with 684,123 cases of constipation and 5,223,378 cases without constipation were included in the meta-analysis. The definition of constipation is different among these studies. The characteristics of the studies and of their participants are presented in Table 1. Among thirteen studies, six were conducted primarily in the United States (8, 17, 21–24), four from Asian countries (14, 25–27), one from Australian (16), and two studies were from European countries (15, 28). Eleven studies included both men and women, two studies included only women. Of these studies included in our meta-analysis, nine were cohort studies (including one nested case-control study) (8, 14, 21–23, 25–28), and the remaining four were cross-sectional studies (15–17, 24). The Newcastle-Ottawa Scale was uniformly applied to assess methodological quality across all thirteen included studies, with each achieving a minimum quality threshold of six points. Comprehensive documentation of bias risk evaluations has been systematically compiled in Table 2.

**Table 1:** Characteristics of included studies in the meta-analysis.

Study (First Author)	Year	Country	Study design	Participants	Definition of constipation	Duration of follow-up (years)	Number with constipation	Number without constipation	Women (%)	Age (years)	Adjustment for confounding variables
Salmoirago-Blotcher et al.	2011	USA	cohort study	postmenopausal women	Defined as “difficulty having bowel movements” over the previous 4 weeks, was rated using a scale ranging from none (did not occur), (mild did not interfere with usual activities), moderate (interfered somewhat with usual activities), or severe (symptom was so bothersome that usual activities could not be performed).	6.9	Mild:18790 Moderate:5391 Severe: 1167	47,699	100.0	63.4	Adjustment for demographics, risk factors, dietary factors, medications, frailty and other psychological variables
Choung et al.	2016	USA	cohort study	community residents	Rome III criteria.	NA	307	2020	NA	61	Adjusted for age and gender.
Honkura et al.	2016	Japan	cohort study	The subjects were all National Health Insurance (NHI) beneficiaries, aged 40–79 years.	Defined as the defecation frequency groups: ≤ 1 time/4 days.	13.3	835	36,158	64.0	59.8	Adjusted for age, sex, body mass index, hypertension, diabetes mellitus, smoking status at baseline, alcohol consumption, education level, time spent walking per day, baseline job status, stress awareness, marital status, fruit and vegetable intake.
Kubota et al.	2016	Japan	cohort study	subjects aged 40 to 79 years, without a history of CVD or cancer.	bowel movement once every 4 or more days	19	Men:316 Women:1861	Men:26346 Women:28738	53.4	57.1	Adjusted for age, history of hypertension, history of diabetes, body mass index, alcohol intake, smoking status, depressive symptoms, perceived mental stress, walking, sports, energy-adjusted dietary fiber intake, living in urban areas and menopausal status for women.
Ma et al.	2016	USA	cohort study	women free from CVD and cancer	Frequency of bowel movements: Every 3–4 days or Every 5 days or less	up to 30	Every 3–4 days:6348 Every 5 days or less:1067	54,264	100.0	48.4	Adjusted for age, ethnicity, menopausal status, smoking status, physical activity, family history of myocardial infarction, baseline history of hypertension, hypercholesterolemia, ulcerative colitis, cholecystectomy, use of multivitamin, aspirin, other nonsteroidal anti-inflammatory drugs, thiazide diuretics, thyroid hormone, alcohol intake, Alternate Healthy Eating Index score, dietary intake of total fiber, total energy intake, body mass index and baseline history of diabetes.
Sumida et al.	2019	USA	cohort study	veterans with an estimated glomerular filtration rate (eGFR) ≥ 60 mL/min/1.73 m^2^	Defined as either having ≥2 prescriptions of laxatives of ≥30-day supply each, that were 60–365 days apart during the baseline period based on information obtained from VA Pharmacy dispensation records; or having at least 2 diagnoses for constipation, as identified by the ICD-9-CM, that were ≥60 days apart.	6.7	237,855	3,121,798	6.8	59.8	Multivariable adjustments for demographics, prevalent comorbidities, medications, and socioeconomic status.
Sundbøll et al.	2020	Denmark	cohort study	constipated patients in contact with the healthcare system, excluded patients with a previous or concurrent inpatient or outpatient diagnosis of any of the study outcomes.	Diagnosed according to the International Classification of Diseases, Eighth Revision (ICD-8) through 1993 and 10th Revision (ICD-10) thereafter	10	83,239	8 32,384	59.0	46.5	Adjusted for age, sex, calendar year, hypothyroidism, hyperthyroidism, pregnancy within 90 days before the index date, depression, Parkinson’s disease, multiple sclerosis, colon, rectal and anal cancer, other gastrointestinal cancers, Crohn’s disease, ulcerative colitis, paralytic ileus, chronic pulmonary disease, valvular heart disease, diabetes mellitus, hypertension, hypercholesterolemia, obesity, chronic kidney disease, liver disease, alcoholism- related disorders, medications associated with constipation and cardiovascular drugs.
Yang et al.	2020	China	cohort study	without cancer, heart disease or stroke at baseline	Frequency of bowel movements: Less than three times a week	10	21,148	373,054	59.1	51.5	Adjusted for sex; level of education; occupation; household income; marital status; family history of certain diseases; smoking status; total physical activity level; alcohol consumption; intake frequency of fresh vegetables, fresh fruit and red meat; BMI (kg/m2); waist circumference (cm); prevalent hypertension and diabetes at baseline.
Peng et al.	2022	USA	cross-sectional study	The National Health and Nutrition Examination Survey (NHANES) database 2005–2010.	The usual or most common stool of BSFS type 1 (separate hard lumps, like nuts) or type 2 (sausage-like, but lumpy), or bowel movements less than 3 times a week was defined as chronic constipation.	NA	1,486	11,986	49.4	47.8	Adjusted for gender, age, race, education, marital status, PIR, BMI, physical activity, smoking status, alcohol, hypertension, dyslipidemia, and cardiotoxic drugs.
Du et al.	2023	USA	cross-sectional study	The data examined were sourced from the National Health and Nutrition Examination Survey (NHANES) for three time intervals: 2005–2006, 2007–2008, and 2009–2010.	Stool traits which were as follows were categorized as constipate: (1) separate hard lumps, like nuts; (2) sausage-like, but lumpy. Constipation was also defined in cases when the frequency of bowel movements was less than thrice a week.	NA	780	7,708	52.30	NA	Adjusted for gender, age (years), race, educational level (less than high school, high school, or above high school), smoking status (never, former, or current) and alcohol consumption (non-drinkers, moderate-drinkers, or heavy drinkers).
Judkins et al.	2023	Australian	cross-sectional study	hospitalized patients aged ≥ 60 years	Constipation (K59.0) was identified according to the International Classification of Diseases, 10th Revision	NA	270,586	270,586	54.6	73.7	Adjusted for age, sex, and cardiovascular risk factors (obesity, smoking, diabetes, sleep apnoea, COPD, kidney disease, endocrine disorders, metabolic disorders, POAD, atrial fibrillation, cardiac arrhythmia), gastrointestinal disorders (irritable bowel syndrome, ulcerative colitis, Crohn’s disease and other gastrointestinal disorders), and metropolitan residence.
Zheng et al.	2024	UK	cross-sectional study	The UK Biobank (UKBB) is a longitudinal cohort consisting of around 500,000 individuals aged between 40 and 69 years in the UK	Constipation was identified through medical records with any encounters of ICD10 code “K59.0.”	NA	23,814	384,540	54.1	56.9	Adjusted for sex, age, Body mass index, Calcium channel blockers, and Standard modifiable cardiovascular risk factors (including hypertension, diabetes, smoking status, and hypercholesterolemia).
Park et al.	2025	Korea	cohort study	Patients undergoing maintenance hemodialysis	Using the total number of prescribed laxatives ≥180 during the 1-year baseline period	5.4	9,133	26,097	41.2	60.1	Adjusted for age, sex, dialysis vintage, comorbidities (diabetes mellitus, hypertension, ischemic heart disease, congestive heart failure, cerebrovascular disease, atrial fibrillation or flutter, and malignancy)

**Table 2:** Results of quality of studies in meta-analysis by Newcastle Ottawa scale (NOS).

Study	Study design	Selection				Comparability	Exposure		
		Adequate definition of cases	Representativeness of the cases	Selection of controls	Definition of controls	Control for important factor *	Ascertain-ment of exposure	Same method of ascertainment for cases and controls	Non-response rate
Salmoirago-Blotcher et al.	cohort study	★	☆	★	★	★★	★	★	★
Choung et al.	cohort study	★	★	★	★	★☆	★	★	★
Honkura et al.	cohort study	★	★	★	★	★★	★	★	★
Kubota et al.	cohort study	★	☆	★	☆	★☆	★	★	★
Ma et al.	cohort study	★	☆	☆	★	★★	★	★	★
Sumida et al.	cohort study	★	☆	★	★	★☆	★	★	★
Sundbøll et al.	cohort study	★	☆	★	★	★★	★	★	★
Yang et al.	cohort study	★	☆	★	★	★☆	★	★	★
Peng et al.	cross-sectional study	★	★	☆	☆	★★	★	★	★
Du et al.	cross-sectional study	★	★	☆	☆	★★	★	★	★
Judkins et al.	cross-sectional study	★	☆	☆	★	★★	★	★	★
Zheng et al.	cross-sectional study	★	★	☆	☆	★☆	★	★	★
Park et al.	cohort study	★	☆	★	★	★☆	★	★	★

^*^ A maximum of 2 stars can be allotted in this category, one for age, the other for other controlled factors.

### 3.2 Constipation and risk of stroke

**Figure 2:**
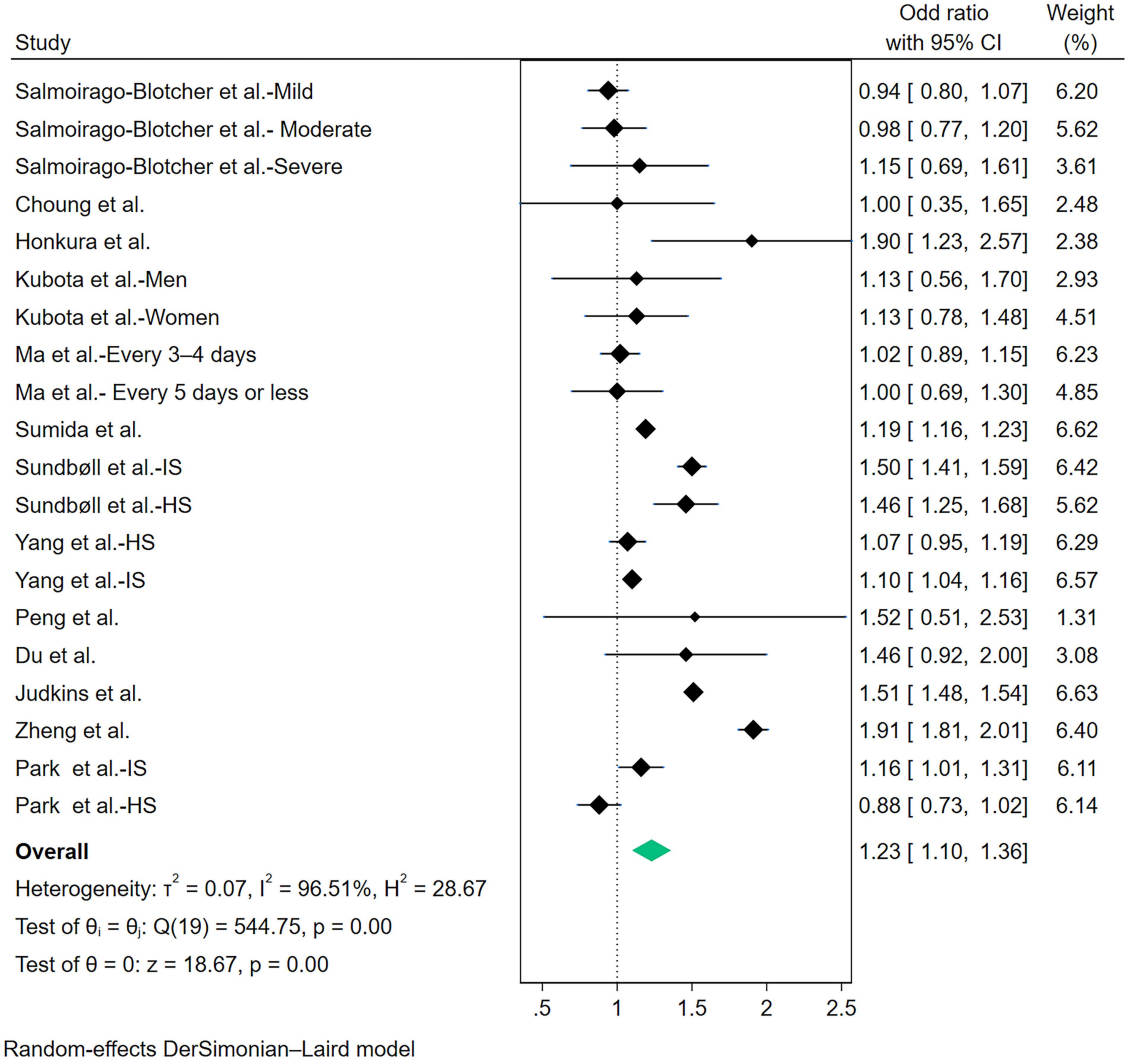
Forest plot of meta-analysis of included studies on the association between constipation and risk of stroke.

**Figure 3:**
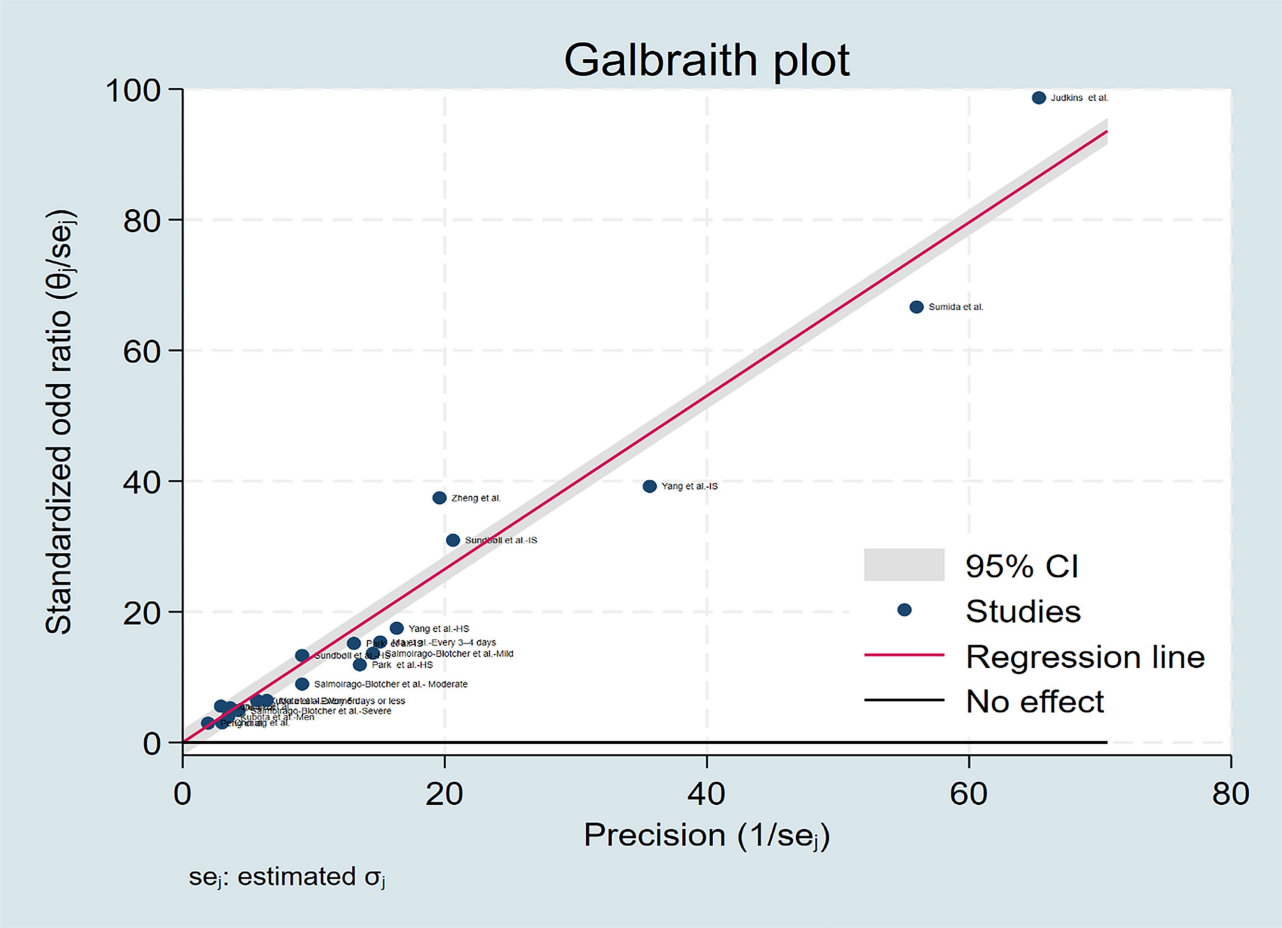
The Galbraith plot regarding the primary source of heterogeneity.

The multivariable adjusted ORs of stroke risk in relation to constipation from individual studies and the combined OR are presented in Figure 2. Some studies do not provide data on constipation and stroke risk at the overall level, so we only extracted data specifically based on disease severity, gender, and stroke subtypes. On the whole, participants with constipation, compared with those without constipation, experienced a significant increased risk of stroke with a pooled odd ratio of 1.23(95% CI: 1.10–1.36). Substantial heterogeneity was observed (*p* < 0.01, *I*^2^ = 96.5%), and the Galbraith plot was conducted to determine the primary source of heterogeneity (Figure 3).

**Figure 4:**
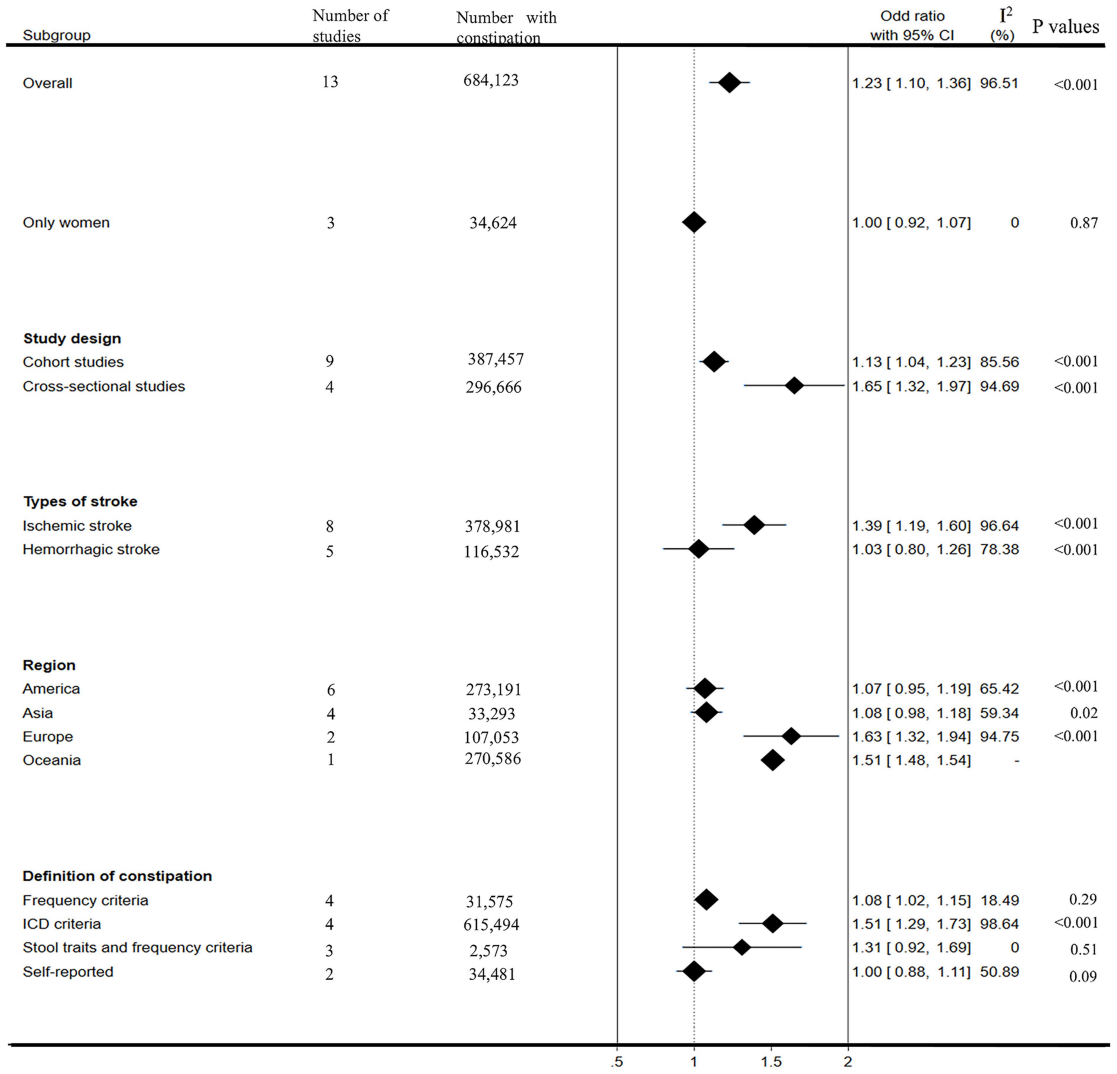
Forest plots of subgroup studies.

Figure 4 presents the subgroup analysis results. In the female populations, our results showed that constipation was not significantly associated with stroke risk (OR: 1.00, 95% CI: 0.92–1.07), although heterogeneity decreased significantly (*I*^2^ = 0%). Considering that the study design may affect the results of our meta-analysis, we further divided it into cohort studies and cross-sectional studies for subgroup analysis. In both subgroups, we observed a strong association between constipation and stroke risk (OR: 1.13, 95% CI: 1.04–1.23, *I*^2^ = 85.56%; OR: 1.65, 95% CI: 1.32–1.97, *I*^2^ = 94.69%; respectively). In subgroup analyses based on stroke type, a significantly increased pooled risk of ischemic stroke for patients with constipation was observed, with an overall OR estimated from the meta-analysis: 1.39(95% CI: 1.19–1.60, *I*^2^ = 96.64%). However, the pooled risk of hemorrhagic stroke did not increase in patients with constipation, and the overall OR estimated was 1.03(95% CI: 0.80–1.26, *I*^2^ = 78.38%). In the subgroup analyses based on region, significant increases in stroke risk were observed in people with constipation in Europe (OR: 1.63, 95% CI: 1.32–1.94, *I*^2^ = 94.75%) and Oceania (OR: 1.51, 95% CI: 1.48–1.54), not in Asia (OR: 1.08, 95% CI: 0.98–1.18, *I*^2^ = 59.34%), Americas (OR: 1.07, 95% CI: 0.95–1.19, *I*^2^ = 65.42%). Considering that different definitions of constipation may affect the results of our meta-analysis, we further conducted a subgroup analysis according to the definition of constipation. Our results showed that patients diagnosed with constipation according to frequency criteria and ICD criteria have a significantly increased risk of stroke (OR: 1.08, 95% CI: 1.02–1.15, *I*^2^ = 18.49%; OR: 1.51, 95% CI: 1.29–1.73, *I*^2^ = 98.64%, respectively). In both subgroups diagnosed with constipation according to stool traits and frequency criteria and self-reported criteria, although heterogeneity was significantly reduced, constipation was not observed to increase stroke risk (OR: 1.31, 95% CI: 0.92–1.69, *I*^2^ = 0%; OR: 1.00, 95% CI: 0.88–1.11, *I*^2^ = 50.89%, respectively).

### 3.3 Publication bias and sensitivity analysis

**Figure 5:**
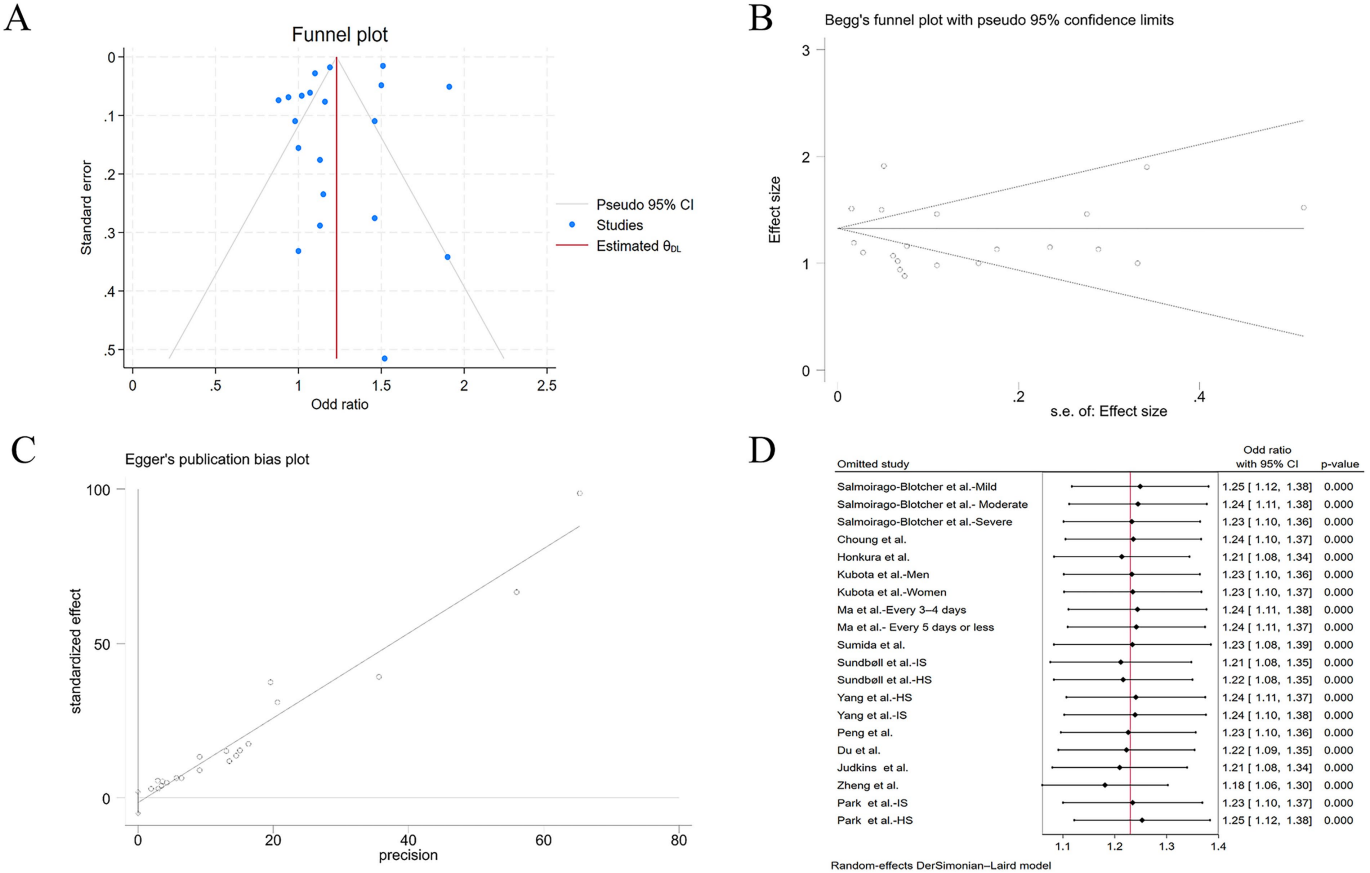
**(A)** Adjusted funnel plot based on the nonparametric trim-and-fill analysis of publication bias; **(B)** evaluation of publication bias by Begg’s funnel plot; **(C)** evaluation of publication bias by Egger’s test; **(D)** sensitivity analysis of the included studies.

To rigorously address potential publication bias in the meta-analysis, we adopted a three-pronged approach: visual inspection of funnel plot symmetry, Begg’s rank correlation method, and Egger’s linear regression test. Following the detection of initial funnel plot asymmetry, we conducted sensitivity analysis using the trim-and-fill method. This procedure can ultimately prove symmetry by incorporating some theoretically missing studies to generate an adjusted funnel plot. But in our meta-analysis, the procedure generated an adjusted funnel plot by incorporating zero imputed studies, and thereby refuted the presence of significant publication bias (Figure 5A). In addition, there was no statistical evidence of publication bias among studies by using Begg’s test (*p* = 0.105) (Figure 5B), and Egger’s test (*p* = 0.364) (Figure 5C). When a single study involved in the meta-analysis was deleted each time, the results of meta-analysis showed that the association between constipation and stroke risk remained significant, indicating that the results of the current meta-analysis were stable (Figure 5D).

### 3.4 Regression analysis

To evaluate some factors that may have affected the results, we conducted a regression analysis of the all included studies (Table 3). Based on regression models studied in constipation patients and control subjects, we found that study design (*p* = 0.001, Adj R-squared = 53.27%), region (*p* = 0.002, Adj R-squared = 48.94%), and gender composition (*p* = 0.043, Adj R-squared = 18.90%) were confounding factors that significantly affected stroke risk. Other risk factors, including category of stroke (*p* = 0.778, Adj R-squared = −5.93%), and definition of constipation (*p* = 0.336, Adj R-squared = −0.12%) did not affect the results.

**Table 3:** Results of meta regression analysis.

Variables	Coefficient (SE)	95% CI	*p*	Adj R-squared
Study design	0.53(0.13)	[0.25, 0.80]	0.001	53.27%
Region	0.20(0.06)	[0.09, 0.32]	0.002	48.94%
Category of stroke	−0.03(0.09)	[−0.22, 0.17]	0.778	−5.93%
Definition of constipation	−0.06(0.06)	[−0.18, 0.06]	0.336	−0.12%
Gender composition	0.30(0.14)	[0.01, 0.59]	0.043	18.90%

SE Standard error, CI Confident interval.

## 4 Discussion

Our updated meta-analysis of 13 published studies confirmed a modest but statistically significant positive association between constipation and risk of stroke. Our results show that patients with constipation have a 1.23 times higher risk of stroke than those without constipation. Given the distinct pathophysiological mechanisms underlying hemorrhagic and ischemic stroke subtypes, we conducted a subgroup analysis. The disease-specific risk profile revealed a statistically significant association between constipation and ischemic stroke (OR = 1.39, 95% CI: 1.19–1.60), whereas no significant relationship was observed in hemorrhagic stroke (OR = 1.03, 95% CI 0.80–1.26). Interestingly, our subgroup analysis revealed a null association between constipation and stroke among women (OR = 1.00, 95%CI 0.92–1.07).

Stroke is the second most common cause of death and the leading cause of disability globally (29). Although significant progress has been made in the prevention and treatment of stroke in the past few decades, the burden will increase significantly due to factors such as population aging, especially in developing countries (29). Strokes manifest as two distinct pathophysiological entities - ischemic and hemorrhagic stroke - that while exhibiting divergent etiological pathways, share overlapping modifiable risk profiles. Both subtypes demonstrate strong epidemiological associations with hypertension, diabetes, smoking, hyperlipidemia, obesity, etc (30). Our results suggest that constipation is associated with the development of stroke. These findings indicate that the pathological characteristics of stroke may be accelerated by constipation, or the two may share etiological pathways.

The observed association between constipation and stroke may be mediated through multiple interrelated biological pathways. Firstly, the gut-brain axis, which serves as a pivotal bidirectional communication axis between the gut and the brain, may plays an important role in the connection between constipation and stroke. Constipation manifests as infrequent defecation, passage of hardened or lumpy stools, straining during elimination, sensation of incomplete evacuation, perception of anorectal obstruction/blockage, and necessity for manual maneuvers to enable defecation (31). Extended colon transit time during constipation may cause alteration of the intestinal microbiota (21). The gut microbiota has a key role in bidirectional interactions in the gut-brain axis, through modulation of central nervous system, immune cells, neuroendocrine cells, and peripheral neurons (32). In the gut-brain axis, migration of intestinal immune cells to the central nervous system may contribute to the pathogeneses of neurological and neurodegenerative diseases, such as stroke (33). Gut microbiota also play a pivotal role in Trimethylamine N-oxide (TMAO) production, augmented macrophage cholesterol accumulation and foam cell formation, all of which promote atherosclerosis progression (34). In addition, the prolonged colonic transit time in constipation may further promote the translocation of proinflammatory cytokines caused by gut bacteria and result in increased inflammatory responses and oxidative stress. So, patients with constipation may sustain a systemic low-grade inflammation state, which accelerates the development of atherosclerosis (35, 36). Secondly, chronic constipation might lead to subsequent persistent increases in blood pressure, through its induction of psychological stress, increased water absorption, and gut dysbiosis (16, 37). Constipation can also cause straining during defecation, during which patients may breathe in a strained manner similar to the Valsalva maneuver (38). This may induce transient blood pressure elevation, potentially triggering stroke occurrence. Third, serotonin (5-hydroxytryptamine, 5-HT) signaling represents a plausible mediating pathway. 5-HT is a key neurotransmitter in the brain-gut axis and is involved in several functions of the gastrointestinal tract including peristaltic reflexes (39). Current evidence remains equivocal regarding the expression of 5-HT increases or decreases in patients with constipation. Some studies suggest that patients with constipation have enhanced synthesis and release of 5-HT (40, 41). And 5-HT, as a vasoconstrictor that facilitates thrombus formation, is associated with the development of atherosclerotic plaques and elevated risks of atherosclerotic cardiovascular disease (42, 43). But a study conducted by Dunlop et al. indicates that relatively low post-prandial plasma 5-HT levels have been detected in patients with constipation-predominant irritable bowel syndrome (44). In generally, acute vascular constriction by 5-HT is usually shared by 5-HT_1B_ and 5-HT_2A_ receptors, except in intracranial arteries which constrict only through 5-HT_1B_ receptors (45). Studies have confirmed that 5-HT-induced vascular constrictions are mediated through 5-HT_1B_ receptors at low 5-HT concentrations (46–48). The serotonin pathway’s implications warrant further investigation.

Noteworthily, our meta-analysis demonstrated a significant correlation between constipation and elevated ischemic stroke risk, whereas no such association for hemorrhagic stroke. We hypothesize that the observed differences may be attributable to the distinct pathogenic mechanisms underlying the two stroke subtypes. The predominant mechanism underlying hemorrhagic stroke is hypertensive small-vessel disease, characterized by the formation of lipohyalinotic microaneurysms that eventually rupture (49). However, the majority of ischemic strokes are thromboembolic in nature, with common sources of embolism including large artery atherosclerosis and cardiac conditions, particularly atrial fibrillation (50). As previously established, chronic constipation contributes to the occurrence and development of atherosclerosis through mechanisms involving gut microbiota dysbiosis and additional biological pathways. Furthermore, Zhang et al. demonstrated that gut microbiota dysbiosis contributes to age-related atrial fibrillation via the lipopolysaccharide- and glucose-mediated activation of the NLRP3-inflammasome pathway (51). A two-sample Mendelian randomization analysis has provided additional evidence supporting a causal association between constipation and atrial fibrillation risk (52). In conclusion, constipation serves as a risk factor for ischemic stroke through multiple pathophysiological mechanisms.

Subgroup analyses demonstrated that constipation was not observed to increase stroke risk in both subgroups diagnosed with constipation according to stool traits and frequency criteria and self-reported criteria. Across three investigations employing stool traits and defecation frequency as diagnostic criteria, constipation conferred a 1.31-fold elevated stroke risk relative to non-constipated counterparts, albeit without statistical significance (8, 17, 24). We believe the underlying reason is that the number of participants was limited in these three studies, whose pooled analytical weight contributed minimally (6.87%) to the aggregate meta-analysis estimate. The two investigations employing self-reported criteria enrolled substantially larger cohorts, collectively contributing 27.68% to the aggregate meta-analytic weight (14, 21). Salmoirago-Blotcher et al. collected information on constipation by self-administered questionnaire. In this study, constipation was defined as “difficulty having bowel movements” over the previous 4 weeks, was rated using a scale ranging from none (did not occur), (mild did not interfere with usual activities), moderate (interfered somewhat with usual activities), or severe (symptom was so bothersome that usual activities could not be performed) (21). Park et al. defined constipation using the total number of prescribed laxatives ≥180 during the 1-year baseline period (14). Self-reported constipation exhibits lower diagnostic accuracy than symptom-based criteria (e.g., defecation frequency or Rome classifications), resulting in inflated prevalence estimates that may attenuate constipation-stroke risk associations.

Interestingly, our meta-analysis results show that constipation is not statistically significantly associated with stroke risk among female participants, and the reasons behind this are worth exploring. We hypothesized that pathophysiological mechanisms underlying the predominant subtype of constipation in female patients play a key important role in this phenomenon. As we know, the clinical spectrum of constipation encompasses three distinct phenotypes: (1) dyssynergic defecation disorder characterized by impaired rectal evacuation, (2) colonic dysmotility manifesting as slow-transit constipation, and (3) normal transit constipation presenting without discernible defecatory dysfunction or delayed colonic transit (53). Epidemiologic data consistently identify female sex as a predisposing factor for chronic constipation development (54). Compared with men, women are more likely to have findings suggestive of a functional defecatory disorder (55). And among women, dyssynergic defecation is the most prevalent subtype and slow transit constipation without dyssynergic defecation is uncommon (56). We postulate that, reduced fecal colonic residence time in dyssynergic defecation patients may confer relative protection against gut microbiota dysbiosis, compared to slow transit constipation cohorts, potentially mediated by diminished microbial fermentation duration. Thus, the reduced probability of gut microbiota dysbiosis may attenuate the association between constipation and stroke risk in women.

Our meta-analysis confirms the association between constipation and stroke risk, which raises interesting questions regarding the potential impact of therapeutic interventions on modifying stroke risk. The Danish population-based study revealed that, compared to the general population cohort, high-intensity laxative use (≥2 prescriptions) was associated with a 3.2-fold increased risk of ischemic stroke, low-intensity users(0–1 prescriptions) was 1.39 (28). Similarly, the US veteran cohort found that, compared to patients without constipation, patients using ≥2 types of laxatives faced a 21% higher stroke risk, patients using one faced a 19% (23). The observed associations potentially arise from two non-exclusive pathways: (1) intensified pharmacotherapy for constipation correlates with greater baseline disease severity, and (2) laxative regimens may inadvertently contribute to stroke pathogenesis through enhanced serotonin bioavailability or laxative-induced dehydration (23). Therapeutic optimization remains paramount, as first-line medical interventions (e.g., fiber supplementation, laxatives) often fail in refractory cases, particularly among patients with functional defecation disorders (e.g., dyssynergia). Management efficacy fundamentally depends on underlying etiology, with an example that biofeedback therapy is the preferred treatment for constipated patients with dyssynergia (57). Future prospective studies should evaluate whether etiology-targeted therapies—including intestinal microbiome modulation via probiotics, biofeedback protocols, or prokinetic agents—mitigate stroke incidence in constipated cohorts.

In interpreting the results, some limitations of this meta-analysis should be considered. First of all, substantial heterogeneity was observed in the present study, which may be related to gender, study type, region, stroke subtype, constipation definition, follow-up time, and so on. We observed a decrease in heterogeneity in subgroup analyses based on sex, region, constipation definition, and study type, which confirmed our hypothesis. We did not observe an association between constipation and stroke risk in studies from Asia and the America, which may be related to confounding factors such as stroke type and gender. However, due to the limited number of studies and the difficulty of obtaining detailed data, we did not perform further subgroup analysis. Notably, in the meta-analysis based on these cohort studies, constipation was still significantly associated with stroke risks, suggesting that our results are relatively reliable. Second, due to the small number of studies included in the subgroup analyses based on stroke subtypes, more studies are needed to determine the reliability of the association between constipation and different stroke subtypes. Third, this is a study-level meta-analysis, not an individual patient meta-analysis, which limits the ability to adjust for confounders at the participant level. Fourthly, our restricted database retrieval (not including Scopus/Embase) and exclusion of some types of literature (conference abstracts/non-English publications) may introduce selection bias through omitted eligible studies. Finally, sex-based subgroup studies are limited and we did not analyze constipation and stroke risks in men, which may inspire future research explorations.

The quality of the studies was assessed via means of the NOS, and all studies included in the analysis had scores ≥6 points. Therefore, the resultant risk estimate resulting from this analysis may also be deemed as fairly stringent estimate. It’s worth noting that these risk estimates demonstrate greater susceptibility to underestimation than overestimation of the true constipation-stroke association magnitude, attributable to preferential adoption of conservative constipation definitions. This is substantiated by subgroup analyses wherein strictly-defined constipation cohorts (ICD criteria/ stool traits and frequency criteria groups) demonstrated elevated stroke risk relative to loosely-defined populations (frequency criteria/ self-reported criteria groups). In future studies, as the number of studies permits, we recommend using universal definitions of constipation whenever possible, such as meeting the Rome IV criteria, to better determine the association between constipation and stroke risk.

## 5 Conclusion

Our meta-analysis demonstrated a significant positive association between constipation and stroke risk. Disease-specific stratification revealed elevated ischemic stroke risk but non-significant hemorrhagic stroke association. Notably, subgroup analyses showed complete risk attenuation in female populations. These findings underscore constipation as a modifiable risk factor in ischemic stroke management. Future studies on the risk of stroke in patients with constipation, and the explanatory factors for this association, are warranted in order to confirm and expand these results.

## Data Availability

The original contributions presented in the study are included in the article/supplementary material, further inquiries can be directed to the corresponding authors.
